# Reliability and Validity of the Serbian Version of the Hidradenitis Suppurativa Quality of Life Questionnaire (HSQoL-24)

**DOI:** 10.3390/jcm15062436

**Published:** 2026-03-22

**Authors:** Milana Marinkovic, Milan Stojičić, Marko S. Jović, Jelena Rakocevic, Zoran Bukumirić, Milana Jurišić, Milan D. Jovanović, Jelena Jeremić, Aleksandar M. Vlahovic, Isidora Vujčić, Nataša Maksimović

**Affiliations:** 1 Clinic for Burns, Plastic and Reconstructive Surgery, University Clinical Center of Serbia, 11000 Belgrade, Serbia; 2 Faculty of Medicine, University of Belgrade, 11000 Belgrade, Serbia; 3 Institute of Histology and Embryology “Aleksandar Đ. Kostić”, 11000 Belgrade, Serbia; 4 Institute for Medical Statistics and Informatics, 11000 Belgrade, Serbia; 5 Institute for Mother and Child Health Care of Serbia, 11000 Belgrade, Serbia; 6 Institute of Epidemiology, Faculty of Medicine, University of Belgrade, 11000 Belgrade, Serbia

**Keywords:** hidradenitis suppurativa, HSQoL-24 questionnaire, quality of life, questionnaire validation, patient-reported outcomes, psychometric properties

## Abstract

**Background:** Hidradenitis suppurativa (HS) is a chronic inflammatory skin disease characterized by painful nodules, abscesses, and sinus tracts in apocrine gland-rich areas, leading to significant impairment in patients’ quality of life (QoL). Persistent symptoms affect physical functioning, psychological well-being, social interactions, and intimate relationships. Therefore, validated disease-specific instruments are essential for accurate QoL assessment in different populations. **Objective:** The aim of this study was to validate the Serbian version of the Hidradenitis Suppurativa Quality of Life Questionnaire (HSQoL-24). **Methods:** The validation process followed established methodological guidelines for psychometric evaluation. The Serbian HSQoL-24 version demonstrated satisfactory psychometric properties. Internal consistency was excellent, with a Cronbach α of 0.907. Test–retest reliability was confirmed using an intraclass correlation coefficient (ICC) of 0.986 for the total score, demonstrating satisfactory reproducibility over time. A strong positive correlation was observed between the HSQoL-24 and Dermatology Life Quality Index (DLQI) total scores (Spearman’s ρ = 0.806, *p* < 0.001), confirming good convergent validity, particularly in domains related to symptoms and feelings, occupational or educational functioning, and interpersonal relationships. Moreover, total HSQoL-24 scores increased significantly with disease severity, indicating the questionnaire’s ability to discriminate between patients with different Hurley stages and confirming its discriminant validity. **Conclusions:** The Serbian version of the HSQoL-24 questionnaire exhibits appropriate internal consistency, test–retest reliability, and convergent and discriminant validity. These results support its use as a reliable and valid disease-specific instrument for assessing QoL in Serbian-speaking patients with HS in both clinical practice and research settings.

## 1. Introduction

Hidradenitis suppurativa (HS) is a chronic inflammatory skin condition characterized by nodules, abscesses, and sinus tracts, with or without secretion, predominantly affecting apocrine gland-rich areas such as the axillae, inguinal, genital, and perianal regions [1,2,3]. The estimated global prevalence of HS ranges from 0.00033% to 4.1%, depending on the population studied, while in Europe it is approximately 0.7% [4,5]. Owing to its nonspecific clinical presentation, HS is frequently misdiagnosed as a bacterial skin infection, resulting in delayed diagnosis and inadequate treatment [6,7]. Consequently, patients often wait 7–10 years for an accurate diagnosis, during which time the disease may progress to severe forms that impair daily functioning [7,8].

Hidradenitis suppurativa has been recognized as one of the dermatological conditions with the greatest negative impact on patients’ quality of life (QoL) compared to other skin diseases [2,9,10,11]. Persistent pain in the affected areas, chronic discharge, unpleasant odor, and itching are typical symptoms [1,9,10,12,13,14]. The long-term presence of these symptoms adversely affects self-esteem, increases the risk of anxiety and depression, and negatively influences intimate relationships, particularly when lesions involve the genital or perianal areas [11,14,15,16,17]. In addition, functional limitations and recurrent disease reduce work capacity, and it is not surprising that a substantial proportion of these patients are unemployed, and their livelihoods are threatened [8,18].

Patient-reported outcome measures (PROMs) enable clinicians to assess patients’ symptoms, functional ability in daily activities, disease-related QoL, and satisfaction with treatment, giving them invaluable information about the overall impact of the disease [11,19,20].

While dermatology instruments, such as the Dermatology Life Quality Index (DLQI), are widely used to assess quality of life in dermatology, they are generic tools and may not fully capture the disease-specific burden of hidradenitis suppurativa [11]. In contrast, disease-specific instruments can provide a more comprehensive assessment of quality of life in this patient population. Therefore, the European Academy of Dermatology and Venereology (EADV) recommends prioritizing HS-specific QoL instruments whenever possible (11). There are several disease-specific questionnaires, such as HIDRAdisk, Hidradenitis Suppurativa Burden of Disease (HSBOD), Hidradenitis Suppurativa Impact Assessment (HSIA), Hidradenitis Suppurativa Quality of Life-24 (HSQoL-24), and Hidradenitis Suppurativa Quality of Life (HiSQOL) [21]. However, their use remains limited due to the lack of validated versions in multiple languages and the demanding process of linguistic and cultural adaptation [21,22,23,24,25,26,27,28].

The HSQoL-24, developed by Marron et al. in 2019, is a specific instrument designed for adult patients with HS and demonstrates robust psychometric properties [29]. The HSQoL-24 questionnaire was specifically developed to assess the multidimensional burden of HS, encompassing physical symptoms, emotional distress, and interpersonal relationships. Compared to other HS-specific questionnaires, HSQoL-24 is easy to administer, requires less than 10 min to complete, and uses a four-week recall period that adequately captures patients’ overall well-being while minimizing the influence of emotional fluctuations. Alongside the original Spanish version of the questionnaire, validated English and Polish versions are also available [22,29,30].

Accurate assessment of quality of life in patients with HS is increasingly important in both dermatology and plastic surgery, given HS’s profound effects on body image, skin appearance, scarring, odor, and overall skin condition [20,21]. Validated disease-specific instruments such as HSQoL-24 are essential for evaluating the effectiveness of medical, surgical, and wound care interventions. Therefore, the development and validation of a Serbian version of the HSQoL-24 questionnaire is necessary to enable standardized QoL in Serbian-speaking patients with HS. Accordingly, the aim of this study is to validate the Serbian version of the HSQoL-24 questionnaire.

## 2. Materials and Methods

### 2.1. Study Design

The investigation was designed as a cross-sectional study and was conducted at the Clinic for Burns, Plastic, and Reconstructive Surgery, University Clinical Center of Serbia, from March to December 2025.

### 2.2. Sample Size

The sample size was determined based on previously reported quality of life scores in patients with hidradenitis suppurativa found in the literature. The calculation assumed a 95% confidence level and a predefined level of precision for estimating mean quality of life values. According to these assumptions, the minimum necessary sample size was calculated to be 63 participants. To satisfy potential missing or incomplete data, the predicted sample size was increased by 20%, yielding a targeted minimum sample of 76 participants.

### 2.3. Patient Selection, Inclusion, and Exclusion Criteria

The study included 79 adult patients ≥ 18 years treated for HS at the Clinic for Burns, Plastic, and Reconstructive Surgery, University Clinical Center of Serbia, and who provided written informed consent to participate. Patients were excluded if they declined participation, were younger than 18 years, or were cognitively unable to complete the questionnaires.

A prospective convenience sampling approach was used, whereby all eligible patients who attended the clinic during the study period and agreed to participate were invited to enroll. The final sample of respondents included in the study was 79.

The study was approved by the Ethics Committee of the Clinic (approval No. 1133, 13 December 2023).

### 2.4. Instruments

Socioepidemiological and clinical data were collected from patients’ medical records and anamnesis. Quality of life was assessed using the Serbian versions of the HSQoL-24 and the DLQI questionnaires [31,32]. All participants completed the questionnaires under identical conditions.

The HSQoL-24 questionnaire consists of 24 items assessing personal, economic, psychosocial, clinical, occupational, and relational aspects of quality of life during the four weeks preceding the questionnaire administration [22]. Responses are recorded on a Likert scale ranging from 0 to 4. Items 6, 17, and 22 are reverse scored. Scores for each Qol domain and a total score are calculated by summing the item responses. To obtain valid scores, all items must be completed. The total score ranges from 0 to 96. To transform the total score into percentages, it is multiplied by the coefficient 1.042. The resultant aggregate for each domain is transformed into percentages by multiplying by predefined coefficients: psychosocial domain by 2.08, economic domain by 25.0, employment domain by 12.5, social interactions domain by 6.25, personal domain by 12.5, and clinical domain by 8.33. Total HSQoL-24 scores are interpreted as follows: scores ≤ 24 indicate no impairment of quality of life, scores of 25 to 31 indicate a mild impairment, scores of 32 to 43 indicate moderate impairment, and scores of 44 to 96 indicate severe impairment of quality of life.

### 2.5. Translation and Cultural Adaptation of the HSQoL-24 Questionnaire

Prior to the validation, the English version of the HSQoL 24 questionnaire was translated into Serbian with the author’s permission. Two independent native Serbian speakers fluent in English performed forward translations, which were reconciled into a single Serbian version. This version was then back-translated into English by a third translator who was blinded to the original questionnaire. Discrepancies between the back-translated and original versions were reviewed and resolved by the research team, which included experts in quality of life research and dermatology.

### 2.6. Pilot Testing

The clarity and comprehensibility of the Serbian version of the HSQoL-24 questionnaire were evaluated through pilot testing involving 10 respondents. Following the respondents’ independent completion of the questionnaire, the researchers conducted brief interviews to identify potential difficulties in completing the questionnaire. The participants in the pilot testing indicated that all questions were straightforward and comprehensible, and they did not require further assistance to complete them. Following pilot testing, the final version of the HSQoL-24 questionnaire was established.

### 2.7. Validation Procedure

The validation of the Serbian version of the HSQoL-24 questionnaire included an assessment of internal consistency, test–retest reliability, and convergent and discriminant validity.

Internal consistency was evaluated using Cronbach’s α coefficient, which measures the homogeneity and interrelatedness of questionnaire items. Cronbach’s α coefficient values range from 0 to 1, with values ≥ 0.70 deemed satisfactory for indicating the internal consistency of the questionnaire.

Test–retest reliability was assessed to evaluate the stability and reproducibility of questionnaire scores over time in participants who did not experience significant clinical changes. This analysis was conducted on a sample of 14 patients who completed the Serbian version of the HSQoL-24 questionnaire twice, with a four-week interval between administrations, during which no significant clinical changes were observed. Reliability was quantified using the intraclass correlation coefficient (ICC). The four-week interval was chosen in accordance with the original version of the questionnaire, which used an interval of 30 ± 10 days [29]. An ICC value greater than 0.75 indicates good test–retest reliability.

Convergent validity of the Serbian version of the HSQoL-24 questionnaire was evaluated by examining the correlation between the overall and domain-specific scores of the HSQoL-24 questionnaire and the corresponding scores of the DLQI questionnaire. Spearman’s correlation coefficient (ρ) was used to assess the strength and direction of associations. Correlation strength was interpreted as very weak (0.00–0.19), weak (0.20–0.39), moderate (0.40–0.59), strong (0.60–0.79), or extremely strong (0.80–1.00). Statistical significance of the correlation was set as *p* < 0.05.

Discriminant validity was assessed by comparing HSQoL-24 scores across patient groups stratified by disease severity according to Hurley stages to determine whether the questionnaire could distinguish between different levels of quality of life impairment.

### 2.8. Additional Instruments and Clinical Assessment

The DLQI is a valid and reliable instrument developed to assess the quality of life in patients with dermatological conditions [33]. Developed in 1994, it has been extensively applied in both clinical practice and scientific research and has been translated and culturally adapted into more than 140 languages [32,33].

The DLQI consists of 10 items addressing symptoms and emotions, leisure activities, daily routines, personal relationships, work or school, and treatment over the preceding week. Items are rated on a scale from 0 to 3, where 0 indicates no impact and 3 indicates a very large impact yielding a total score ranging from 0 to 30. Elevated score values indicate a greater deterioration in quality of life. Total scores are interpreted as follows: scores ≤ 1 indicate no impact on QoL, 2–5 mild impairment, 6–10 moderate impairment, 11–20 very large impairment, and 21–30 extremely large impairment [33].

Disease severity was assessed using the Hurley staging system, which is the most prevalent method for determining disease severity [3]. Patients whose clinical assessment indicates the existence of one or more nodes, without fistulas, sinus tracts, or scar tissue, were classified as having a Hurley I stage. Patients with recurrent abscesses accompanied by formed fistulas, sinus tracts, or localized scar formations were classified as Hurley II stage, whereas those with fistulas, sinus tracts, and scar formations diffusely affecting the entire region were classified as Hurley III stage.

### 2.9. Statistical Analysis

Descriptive statistics were used to describe the study population, including means with standard deviations, medians with ranges (for numerical variables), and numbers and percentages (for categorical variables). The distribution of variables was assessed using the Shapiro–Wilk test. The Cronbach α coefficient was used to measure the questionnaire’s internal consistency. The Spearman correlation test was performed to identify the association between the questions and the correlations with DLQI. The Kruskal–Wallis test and post hoc Dunn test were employed for discriminant validity evaluation. The questionnaire’s test–retest reliability was assessed using the ICC. The data were analyzed using the statistical software package SPSS Statistics, version 23.0 for Windows (Armonk, New York, NY, USA), as well as the R programming language (version 4.5.2; R Foundation for Statistical Computing, Vienna, Austria). Statistical significance was defined as *p* < 0.05.

## 3. Results

A total of 79 patients were included in the study; 74.7% were male and 25.3% were female. The median age of the participants was 40 years (range: 18–73 years). According to the Hurley staging system for disease severity, 12 patients (15.2%) were classified as Hurley stage I, 29 patients (36.7%) were classified as Hurley stage II, and 38 patients (48.1%) as Hurley stage III, representing the most severe clinical presentation. The median number of affected anatomical areas was 3 (range: 1–10).

The median disease duration was 5 years (range: 4 months–32 years), while the median interval from symptom onset to definitive diagnosis was 2 years (range: 1 month–30 years). All patients included in the study underwent surgical treatment for HS. Table 1 summarizes the epidemiological and clinical characteristics of the patients included in the questionnaire validation process.

**Figure 1:**
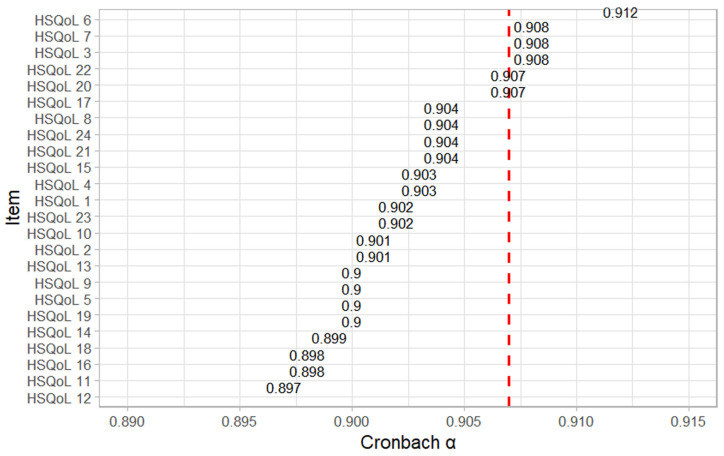
Cronbach’s α values when single items were omitted.

Cronbach’s α coefficient was 0.907, indicating excellent internal consistency. Analysis of the Cronbach α demonstrated that the exclusion of individual items resulted in only minimal changes to the overall coefficient (Figure 1). The lowest Cronbach α value was observed following the exclusion of item 12 (α = 0.897), whereas the highest value was obtained when items 3, 7, or 22 were excluded (α = 0.908 in each case). These findings suggest that no individual item adversely affected the internal consistency of the questionnaire and confirm that the HSQoL-24 demonstrates a high level of internal reliability.

Table 2 presents the results of the test–retest reliability analysis of the Serbian version of the HSQoL-24 questionnaire. The ICC for the total score was 0.986, indicating adequate reproducibility. Statistically significant test–retest reliability was also observed across all questionnaire domains. The lowest ICC was seen in the personal domain (ICC = 0.573, *p* = 0.013), whereas the highest ICC was recorded in the employment domain (ICC = 0.896, *p* < 0.001).

**Figure 2:**
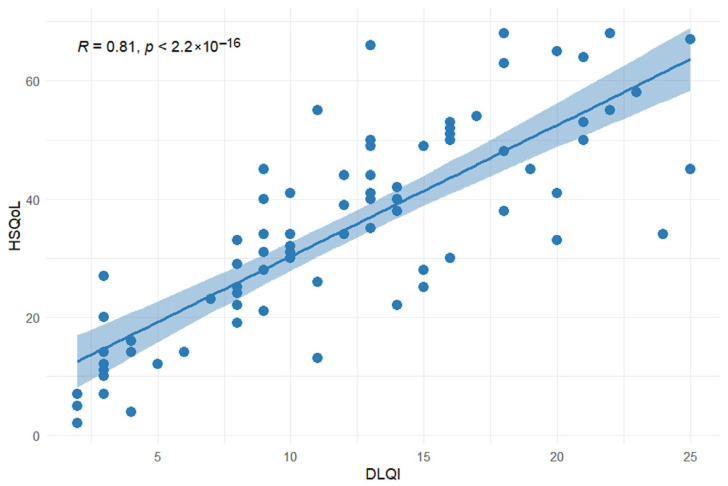
Convergent validity of the HSQoL-24 and DLQI questionnaires.

Convergent validity of the HSQoL-24 questionnaire was evaluated by examining its correlation with the DLQI questionnaire using Spearman’s correlation coefficient. The findings indicated a strong positive correlation between the total scores of the HSQoL-24 and DLQI questionnaires (Spearman’s ρ = 0.806, *p* < 0.001) (Figure 2), indicating that both instruments assess a similar construct of quality of life.

Further domain-specific correlation analysis was performed between the HSQoL-24 and DLQI questionnaires. The DLQI domain concerning symptoms and feelings showed moderate to strong correlation with the HSQoL-24 psychosocial domain (ρ = 0.559; *p* < 0.001) and the HSQoL-24 clinical domain (ρ = 0.475; *p* = 0.001).

The DLQI domain related to work or study demonstrated a moderate correlation with the HSQoL-24 employment domain (ρ = 0.347; *p* = 0.002). The DLQI personal relationships domain showed a strong positive correlation with the HSQoL-24 social interaction domain (ρ = 0.609; *p* < 0.001); however, its correlation with the HSQoL-24 personal domain was not statistically significant (ρ = 0.098; *p* = 0.389). Overall, the HSQoL-24 questionnaire demonstrated adequate convergent validity with the DLQI questionnaire, particularly in domains related to symptoms and feelings, occupational or educational functioning, and interpersonal relationships.

Discriminant validity of the Serbian version of the HSQoL-24 questionnaire was assessed by comparing scores across different Hurley stages of the disease severity. The results demonstrated a statistically significant increase in total HSQoL-24 scores with increasing disease severity (Hurley stage I: 14.0 [2.0–38.0], Hurley stage II: 31.0 [7.0–68.0], Hurley stage III: 46.5 [13.0–68.0]; *p* < 0.001). These findings indicate that the HSQoL-24 questionnaire effectively discriminates between patients with varying levels of disease severity, thereby confirming its discriminant validity. A similar pattern was observed when analyzing the scores associated with certain domains across Hurley stages (Table 3).

## 4. Discussion

This study presents the first validation of the hidradenitis suppurativa-specific HSQoL-24 questionnaire in the Serbian language. The obtained results demonstrate favorable psychometric properties of the Serbian version and are consistent with findings from previously validated versions in other languages [22,29,30].

The Cronbach α coefficient for the Serbian version of the questionnaire was 0.907, demonstrating excellent internal consistency. Analysis of individual items showed that the exclusion of any single question would result in minimal variations in the Cronbach α value. The lowest Cronbach α coefficient was 0.897, while the highest was 0.912, confirming that there were no items that could compromise the reliability of the instrument. These findings indicate that the Serbian version of the questionnaire is both homogeneous and reliable for use in clinical practice. The internal consistency observed in this study is comparable to that reported for the Spanish and Polish versions (Cronbach α = 0.908 and 0.920, respectively) and was slightly higher than that reported for the English version (Cronbach α = 0.866) [22,29,30].

The test–retest reliability analysis of the Serbian version of the HSQoL-24 questionnaire demonstrated high stability of responses between the two administrations of the questionnaire. The total HSQoL-24 score showed an excellent intraclass correlation coefficient (ICC = 0.986; 95% CI 0.957–0.996; *p* < 0.001), indicating outstanding repeatability of the answers. The ICC value indicates that the respondents’ answers were consistent across the two-time measurements, thereby confirming the instrument’s stability over time. Although the derived ICC values demonstrate satisfactory reproducibility, the small sample size may compromise the reliability of these estimates and should be interpreted with caution. Future studies involving larger participant cohorts are needed to provide more robust evaluations of stability over time. Furthermore, the evaluation of specific domains indicated that psychosocial (ICC = 0.869), economic (ICC = 0.846), employment (ICC = 0.896), and social interaction (ICC = 0.854) domains achieved satisfactory test–retest reliability. These results are consistent with the original Spanish validation study, which reported an ICC of 0.842 for total score and domain-specific ICC values ranging from 0.682 to 0.872 [29]. Similarly, the Polish version verified strong test–retest reliability (ICC = 0.908 for the overall score) [30]. In contrast, test–retest reliability was not assessed for the English version [22].

In the present study, the lowest ICC values were observed in the clinical (ICC = 0.673) and personal (ICC = 0.573) domains. This finding may reflect the subjective nature of these domains, which can fluctuate over time depending on patients’ emotional state and personal circumstances. The slightly lower ICC observed in the personal domain may indicate the dynamic character of personal perceptions and social interactions, which might vary even in clinically stable patients. Similar variability has been documented in various quality of life measures assessing subjective psychosocial aspects. Additionally, a similar situation was reported in the original Spanish validation, and slightly higher ICC values were observed for these domains (ICC for clinical domain = 0.702 and ICC for personal domain = 0.718) [29]. The Serbian version of the HSQoL-24 questionnaire demonstrated high test–retest reliability, comparable to that reported in other language validations, including the original version of the questionnaire. Exploratory or confirmatory factor analysis was not conducted in this study due to the HSQoL-24 questionnaire having an internationally recognized factor structure established during its original development and validation. The main objective of the current study was to test the reliability and validity of the Serbian translation, rather than to reexamine the underlying factor structure.

Convergent validity of the Serbian version of the HSQoL-24 questionnaire was assessed by examining its correlation with the DLQI questionnaire, a widely used dermatology-specific instrument assessing similar quality of life domains. A robust correlation between the overall scores of the two questionnaires was demonstrated (ρ = 0.806, *p* < 0.001), suggesting that both instruments measure the same construct of quality of life. This correlation was stronger than those reported in previous validation studies [22,29,30]. The Polish version of the HSQoL-24 questionnaire demonstrated a Spearman coefficient of 0.579, while the English and Spanish versions demonstrated coefficients of 0.690 and 0.698, respectively [22,29,30].

Domain-level analysis revealed the expected convergence patterns between the HSQoL-24 and DLQI questionnaires. The strongest associations were observed between psychosocial and social domains, highlighting the substantial emotional burden and stigma associated with HS. Moderate association was observed between the clinical and occupational domains, although a weak correlation was found between the personal domain of the HSQoL-24 and the DLQI. This discrepancy could originate from the greater specificity of the HSQoL-24, which differentiates personal and social functioning more precisely, whereas the DLQI provides a more generalized categorization.

Krajewski et al. further examined convergent validity by comparing the Polish version of the HSQoL-24 with another HS-specific instrument, the HiSQoL, revealing a slightly weaker correlation compared to that observed with the DLQI questionnaire (ρ = 0.559 vs. ρ = 0.579) [30]. On the other hand, Marron et al. assessed the correlation between the HSQoL-24 and the Skindex-29 questionnaire and found a strong positive correlation for both the Spanish (ρ = 0.90) and English (ρ = 0.869) versions) [22,29]. The findings demonstrate that the HSQoL-24 questionnaire demonstrates robust convergent validity with dermatological PROMs and other instruments specifically designed for assessing the QoL of individuals with HS. Comparable domain patterns observed in other HSQoL-24 validation studies confirm that the instrument consistently assesses patients’ quality of life, especially in aspects pertaining to symptoms, relationships, and daily functioning [22,29,30]. The findings support the HSQoL-24 as an effective and sensitive tool for evaluating the disease’s influence on the QoL of patients with HS.

The Serbian version of the HSQoL-24 questionnaire differentiates patients across different disease stages, as seen by the total score of the questionnaire rising with advancing Hurley stage, thereby confirming the questionnaire’s discriminant validity.

Although HS is reported to be more prevalent in women, male patients predominated in our cohort. This likely reflects the characteristics of the study population, as all participants were recruited from a tertiary center, where patients with more severe disease are referred for surgical treatment.

This study demonstrated that the Serbian translation of the HSQoL-24 questionnaire allows a comprehensive assessment of quality of life, including domain-specific evaluation. The acceptable psychometric properties of the questionnaire suggest its applicability in routine clinical practice by physicians, as well as in scientific research, to assess the effects of treatment modalities, as well as to identify individuals whose quality of life has been most significantly compromised by the disease. Validation of the HSQoL-24 questionnaire in Serbian will contribute to the international standardization of quality of life data in patients with HS and facilitate a better understanding of quality of life perceptions in these patients, both locally and internationally.

Beyond its psychometric robustness, the HSQoL-24 questionnaire has considerable practical value in both dermatology and cosmetic dermatology [34]. HS substantially influences visible skin features, including scarring, pigmentation, odor, and chronic inflammation, which directly affect self-perception, aesthetic concerns, and psychosocial functioning [21]. The validated version of the HSQoL-24 questionnaire enables clinicians to systematically evaluate patient-reported outcomes following various therapeutic modalities. Moreover, its detailed domain structure allows clinicians and researchers to identify which aspects of quality of life are most affected, thus supporting individualized treatment planning.

Despite being the first study to validate an HS-specific quality of life questionnaire in the Serbian language, this research has certain limitations. The primary limitation is the relatively small sample size derived from a single tertiary care center, which may limit the generalizability of the findings and introduce selection bias. Further multicenter studies are needed to confirm the generalizability and long-term stability of the instrument. Secondly, the severity of the HS in this study was evaluated utilizing the Hurley classification system, which is commonly used in clinical practice, especially in surgical contexts. Nonetheless, we recognize that Hurley staging is a relatively simple classification and fails to comprehensively reflect inflammatory activity in comparison to other scoring systems such as the International Hidradenitis Suppurativa Severity Score System (IHS4). In addition, the responsiveness of the questionnaire to treatment effects was not evaluated, since the present study was designed as a cross-sectional validation study. Future longitudinal studies are needed to assess the sensitivity of the Serbian HSQoL-24 to clinical changes following therapeutic interventions.

## 5. Conclusions

The Serbian version of the HSQoL-24 questionnaire demonstrates acceptable internal consistency, strong test–retest reliability, and satisfactory convergent and discriminant validity. This disease-specific questionnaire captures multiple dimensions of quality of life in patients with HS, making this PROM valuable and usable in both clinical practice and scientific research.

## Figures and Tables

**Table 1:** The characteristics of patients included in the study.

Variable	N (%)
Gender	
Men	59 (74.7)
Women	20 (25.3)
Age (median, range)	40 (18–73)
Disease severity	
Hurley I	12 (15.2)
Hurley II	29 (36.7)
Hurley III	38 (48.1)
Number of affected areas (median, range)	3 (1–10)
Disease duration (median, range) age	5 (0.4–32)
Diagnostic delay (median, range) age	2 (0.1–30)
Surgical treatment	
Yes	79 (100%)
No	0 (0%)

**Table 2:** Test–retest reliability for the HSQoL-24 questionnaire.

	ICC	95% CI	*p*
HSQoL-24 total score	0.986	0.957–0.996	<0.001
HSQoL-24 psychosocial domain	0.869	0.642–0.956	<0.001
HSQoL-24 economic domain	0.846	0.587–0.948	<0.001
HSQoL-24 employment domain	0.896	0.709–0.965	<0.001
HSQoL-24 social interaction domain	0.854	0.607–0.951	<0.001
HSQoL-24 clinical domain	0.673	0.243–0.882	0.003
HSQoL-24 personal domain	0.573	0.083–0.840	0.013

ICC—Intraclass correlation coefficient, CI—confidence interval.

**Table 3:** The discriminant validity of the HSQoL-24 questionnaire.

	Hurley I	Hurley II	Hurley III	*p* Value	Hurley I vs. Hurley II	Hurley I vs. Hurley III	Hurley II vs. Hurley III
HSQoL-24 total score	14.0 (2.0–38.0)	31.0 (7.0–68.0)	46.5 (13.0–68.0)	<0.001	0.007	<0.001	<0.001
HSQoL-24 total percent	14.59 (2.08–39.60)	32.30 (7.30–70.86)	48.46 (13.555–70.86)	<0.001	0.008	<0.001	<0.001
HSQoL-24 psychosocial domain	14.56 (0–47.84)	31.20 (8.32–74.588)	50.96 (8.32–97.76)	<0.001	0.011	<0.001	0.008
HSQoL-24 employment domain	12.5 (0–50.0)	37.5 (0–100.0)	62.5 (0–100.0)	<0.001	0.057	<0.001	0.023
HSQoL-24 social interaction domain	12.5 (0–37.5)	25.0 (0–75.0)	53.1 (12.5–75.50)	<0.001	0.212	<0.001	0.001
HSQoL-24 personal domain	16.66 (0–33.23)	24.99 (0–66.64)	33.32 (0–91.63)	0.004	0.296	<0.001	0.400
HSQoL-24 economic domain	0 (0–50.0)	25 (0–75.0)	50 (0–100)	0.023	1.000	0.059	0.097
HSQoL-24 clinical domain	0 (0–50.0)	12.5 (0–50.0)	25 (0–100.0)	< 0001	0.091	<0.001	0.068

Values are presented as median (range). *p* values were calculated using the Kruskal–Wallis test with post hoc Dunn test.

## Data Availability

The data presented in this study are available on request from the corresponding author due to ethical restrictions.
